# Early Serum Calcium Assessment as a Cost-Effective Tool in Risk Stratification of Acute Pancreatitis

**DOI:** 10.7759/cureus.92539

**Published:** 2025-09-17

**Authors:** Aakshaay Rastogi, Nitin Gupta, Esha Chaudhary, Akansha Aggarwal, Vikram Rajput

**Affiliations:** 1 Department of General Medicine, Maharishi Markandeshwar Institute of Medical Sciences and Research, Mullana, IND

**Keywords:** acute pancreatitis, bisap score, hypocalcemia, prognostic marker, serum calcium

## Abstract

Background

Acute pancreatitis is a common gastrointestinal emergency that ranges from mild, self-limiting inflammation to severe disease with multi-organ dysfunction and high mortality. Traditional prognostic scoring systems such as Bedside Index for Severity in Acute Pancreatitis (BISAP) and CT Severity Index (CTSI) are useful but often complex. Biochemical markers like serum calcium may provide a simple and accessible alternative for early risk stratification.

Objective

To evaluate the role of serum calcium as a prognostic marker in patients with acute pancreatitis by assessing its correlation with BISAP score, CTSI, duration of hospital stay, need for surgical intervention, and clinical outcomes, including mortality.

Methods

This study was conducted over 18 months at a tertiary care center. Fifty adult patients (≥18 years) with newly diagnosed acute pancreatitis were enrolled. Serum calcium levels measured within 24 hours of admission were analyzed in relation to BISAP score, CTSI, hospital stay, and clinical outcomes. Data were analyzed using the chi-square test, independent t-test, and Pearson’s correlation. A p-value <0.05 was considered statistically significant.

Results

The mean age of patients was 37.9 ± 13.05 years, with a male predominance (66%, n = 33). Gallstones (58%, n = 29) were the most common etiology. Hypocalcemia (<8.5 mg/dL) was observed in 58% (n = 29) of patients. The mean duration of hospital stay was 12.64 ± 4.09 days. The overall mortality rate was 18%, with 82% of patients discharged after recovery. Lower calcium levels were significantly associated with higher CTSI grades (p = 0.001), prolonged hospital stay (r = -0.694, p = 0.001), higher BISAP scores (r = -0.684, p = 0.001), and mortality (p = 0.001). Patients requiring surgical intervention also had significantly lower calcium levels (p = 0.005).

Conclusion

Hypocalcemia is strongly correlated with disease severity, adverse outcomes, and prolonged hospitalization in acute pancreatitis. Early serum calcium measurement within 24 hours of admission may serve as a simple, cost-effective prognostic tool for clinical decision-making.

## Introduction

Acute pancreatitis is an abrupt inflammatory disorder of the pancreas that may progress to systemic involvement and multi-organ dysfunction. It represents one of the most common clinical emergencies encountered in gastroenterology and critical care worldwide. In the United States, the annual incidence of acute pancreatitis is approximately 40 cases per 100,000 population, accounting for more than 300,000 hospital admissions and 20,000 deaths, with an estimated economic burden exceeding USD 2.2 billion [1]. Pancreatitis manifests in two principal forms: acute and chronic. The acute form is histologically characterized by acinar cell necrosis accompanied by parenchymal inflammation [2].

In India, the prevalence of acute pancreatitis is relatively lower but remains clinically significant, estimated at 7.9 cases per 100,000 population, with a nearly equal distribution between males and females [3]. Although nearly 80% of acute pancreatitis cases are mild to moderate and self-limiting, approximately 20% progress to severe acute pancreatitis, which is associated with serious complications, including shock, persistent organ failure, and pancreatic necrosis [4]. According to the 2012 revised Atlanta classification, severe acute pancreatitis is defined as pancreatitis accompanied by organ failure persisting for more than 48 hours, with an associated mortality risk ranging from 20% to 50% [5].

The diagnosis of acute pancreatitis is established when at least two of the following three criteria are met: characteristic epigastric pain, often radiating to the back; serum amylase or lipase levels elevated to more than three times the upper limit of normal; and imaging findings consistent with pancreatitis. Indicators of severity include the presence of systemic inflammatory response syndrome (SIRS), elevated blood urea nitrogen (>22 mg/dL), and hematocrit levels exceeding 44% [6]. Imaging modalities, including transabdominal ultrasonography, contrast-enhanced computed tomography (CECT), MRI, and endoscopic ultrasound, play a crucial role in detecting complications, identifying underlying etiologies, and assessing pancreatic necrosis [7].

Severity scoring systems, such as the Ranson criteria, Bedside Index for Severity in Acute Pancreatitis (BISAP) score, Acute Physiology and Chronic Health Examination (APACHE) II, and the Balthazar CT Severity Index (CTSI), are valuable tools for risk stratification and guiding early management in patients with acute pancreatitis. For example, the BISAP score incorporates five variables: blood urea nitrogen >25 mg/dL, impaired mental status, age >60 years, presence of pleural effusion, and SIRS to predict mortality in acute pancreatitis [7].

Despite these advancements, biochemical markers provide a simpler and more rapid alternative to complex scoring systems. Among these, serum calcium has gained particular attention. Calcium, an essential mineral involved in cellular signaling, vascular regulation, and muscular function, has been shown to correlate inversely with the severity of acute pancreatitis. Hypocalcemia, defined as a serum calcium level below 8.5 mg/dL, is a frequent finding in acute pancreatitis and constitutes one of the parameters in Ranson’s criteria [8]. The pathogenesis of hypocalcemia in acute pancreatitis may involve mechanisms such as fat saponification, hypomagnesemia, or hypoparathyroidism. Notably, hypocalcemia occurs more frequently in severe acute pancreatitis (86%) than in mild disease (39%) and serves as an independent predictor of mortality, not only in acute pancreatitis but also in comorbid conditions such as chronic kidney disease and heart failure [4].

Given its prognostic potential, serum calcium measured within the first 24 hours of hospitalization may serve as a simple, accessible, and cost-effective indicator of disease severity in acute pancreatitis. Although it cannot substitute for comprehensive scoring systems, early calcium assessment may aid in patient triage and guide clinical decision-making. Accordingly, the present study aims to evaluate the role of serum calcium as a prognostic marker in patients with acute pancreatitis.

## Materials and methods

Study design and setting

This prospective, observational, hospital-based study was conducted in the Department of General Medicine at Maharishi Markandeshwar Institute of Medical Sciences and Research (MMIMSR), Mullana, Ambala, India, over a period of 18 months (2023-2025), after obtaining approval from the Institute Ethics Committee (Approval No. IEC-2868). The study population comprised adult patients (>18 years) presenting to the outpatient department or emergency services who were subsequently admitted to the general medicine wards or ICU with a new diagnosis of acute pancreatitis.

Sample size

The sample size was calculated based on the estimated prevalence of acute pancreatitis in India, reported to be approximately 2.6% [9]. Using a confidence level of 95% and a precision of 5%, the calculated minimum required sample size was 39. To account for possible dropouts and enhance statistical power, a total of 50 patients were enrolled in the study.

Inclusion and exclusion criteria

The study included adult patients (≥18 years) presenting with a new diagnosis of acute pancreatitis. The diagnosis was established when at least two of the following three standard criteria were met: characteristic epigastric pain, often radiating to the back; serum amylase or lipase levels elevated to more than three times the upper limit of normal; and imaging findings consistent with acute pancreatitis. Patients with known risk factors, such as alcohol use, abdominal trauma, or gallstone disease, were considered eligible. Written informed consent was obtained from all participants prior to enrollment. Patients were excluded if they had a prior history of chronic pancreatitis or recurrent episodes of acute pancreatitis. Additional exclusion criteria included known chronic kidney disease, parathyroid disorders, pancreatic malignancies, ongoing chemotherapy, or current use of calcium supplements, as these factors could influence serum calcium levels and confound study outcomes.

Data collection procedure

Primary data were collected by the principal investigator through patient interviews, clinical examinations, laboratory investigations, and imaging studies. Serum total calcium was measured within the first 24 hours of admission. Albumin-corrected calcium and ionized calcium values were not assessed in this study. These values were subsequently analyzed in relation to the patient’s BISAP score, CTSI, duration of hospitalization, and clinical outcomes, including mortality.

Bedside Index for Severity in Acute Pancreatitis (BISAP)

The scores were calculated within 24 hours of admission using five parameters: blood urea nitrogen >25 mg/dL; impaired mental status (Glasgow Coma Scale <15); age >60 years; presence of pleural effusion on imaging; and SIRS. Each variable was assigned one point, with the total score ranging from zero to five. Higher scores corresponded to increasing mortality risk: zero to one (low risk, mortality <1%), two to three (moderate risk, mortality ~2-10%), and four to five (high risk, mortality >15%).

CT Severity Index (CTSI)

The CTSI was determined using CECT findings. Pancreatic inflammation and peripancreatic changes were graded from zero to four, while pancreatic necrosis was scored from zero to six, yielding a maximum of 10 points. The overall score was categorized as mild (zero to three), moderate (four to six), or severe (seven to 10). Higher CTSI scores indicated greater disease severity and risk of complications.

Statistical analysis

Data were compiled using Microsoft Excel (Microsoft Corporation, Redmond, Washington, United States) and analyzed using IBM SPSS Statistics for Windows, Version 26 (Released 2018; IBM Corp., Armonk, New York, United States). Continuous variables were presented as means with standard deviations (SD), while categorical variables were expressed as frequencies and percentages. The independent t-test was employed to compare means between two groups, and one-way ANOVA was used for comparison across more than two groups. The chi-square test was used for categorical variables, while Pearson’s correlation coefficient assessed relationships between continuous variables. A p-value <0.05 was considered statistically significant.

## Results

A total of 50 patients with acute pancreatitis were included in the study. The mean age of the study population was 37.92 ± 13.05 years. There was a male predominance, with 66% males and 34% females. The most common etiology was gallstone disease, observed in 58% of cases, followed by alcohol consumption in 30%, and other causes in 12% (Table 1).

**Table 1:** Mean age, gender distribution, and etiology of patients.

Variable	Domain	Value
Mean age		37.92 ± 13.05 years
Gender	Male	33 (66%)
	Female	17 (34%)
Etiology	Gallstone	29 (58%)
	Alcohol	15 (30%)
	Other	6 (12%)

All patients had elevated serum amylase levels, with a mean of 1017.07 ± 479.49 U/L. Similarly, serum lipase was elevated in 98% of patients (mean: 1276.65 ± 526.27 U/L), with only one patient (2%) falling within the normal range. Serum calcium levels showed a mean value of 7.73 ± 1.20 mg/dL. Hypocalcemia (<8.5 mg/dL) was observed in 58% of patients, while 38% had normal calcium levels, and 4% exhibited hypercalcemia (Table 2).

**Table 2:** Biochemical profile of patients.

Variable	Domain	Value
Amylase	Mean value	1017.07 ± 479.49 U/L
	Below normal (<25)	0
	Normal (25-115)	0
	Above normal (>115)	50 (100%)
Lipase	Mean value	1276.65 ± 526.27 U/L
	Below normal (<74)	0
	Normal (74-393)	1 (2%)
	Above normal (>393)	49 (98%)
Calcium	Mean value (mg/dl)	7.73 ± 1.20 mg/dL
	Below normal (<8.5 mg/dl)	29 (58%)
	Normal (8.5-10.5 mg/dl)	19 (38%)
	Above normal (>10.5 mg/dl)	2 (4%)

Based on the CTSI, 20% of patients were classified as having mild disease, 64% as moderate, and 16% as severe. Most patients (60%) were managed conservatively, while 40% required surgical intervention. The mean duration of hospital stay was 12.64 ± 4.09 days. The overall mortality rate was 18%, with 82% of patients discharged after recovery. BISAP scores revealed that the majority of patients had scores between two and three (28% and 32%, respectively), while 14% had a score of zero. Scores of four and five, indicating more severe disease, were observed in 18% and 2% of patients, respectively. These findings reflect a distribution of disease severity and clinical outcomes consistent with the prognostic utility of both CTSI and BISAP scoring (Table 3).

**Table 3:** CT Severity Index, intervention, outcomes, hospital stay duration, and BISAP score. BISAP: Bedside Index for Severity in Acute Pancreatitis

Variable	Domain	Value
CT Severity Index	Mild (0-3)	10 (20%)
	Moderate (4-6)	32 (64%)
	Severe (7-10)	8 (16%)
Intervention	Conservative	30 (60%)
	Surgical	20 (40%)
Outcomes	Death	9 (18%)
	Discharge	41 (82%)
Hospital stays	Mean duration	12.64 ± 4.09 days
BISAP score	0	7 (14%)
	1	3 (6%)
	2	14 (28%)
	3	16 (32%)
	4	9 (18%)
	5	1 (2%)

Patients with mild acute pancreatitis demonstrated the shortest mean hospital stay (7 ± 0.67 days). In contrast, those with moderate and severe forms experienced significantly longer durations, averaging 12.72 ± 1.84 days and 19.38 ± 2.07 days, respectively. This progressive increase in hospitalization with higher disease severity highlights a strong positive correlation between CTSI scores and length of stay. The findings underscore the greater clinical burden and the requirement for prolonged medical management in patients with more severe disease (Table 4).

**Table 4:** CT Severity Index and mean duration of hospital stay.

Variable	Domain	Mean duration of hospital stay
CT Severity Index	Mild	7 ± 0.67 days
	Moderate	12.72 ± 1.84 days
	Severe	19.38 ± 2.07 days

A statistically significant association was observed (p = 0.001). Among patients with hypocalcemia (serum calcium <8.5 mg/dL), the majority had moderate (n = 20) or severe (n = 8) disease. In contrast, normocalcemic patients (8.5-10.5 mg/dL) were predominantly in the mild (n = 7) and moderate (n = 12) categories, while all patients with hypercalcemia (>10.5 mg/dL) had only mild disease. These results suggest that lower serum calcium levels are significantly associated with increased disease severity as assessed by CTSI (Table 5).

**Table 5:** Association of calcium levels with CT Severity Index. *Legend: Data analyzed using chi-square test. p <0.05 indicates statistical significance.

CT Severity Index	Below normal (Ca <8.5 mg/dL)	Normal (Ca 8.5-10.5 mg/dL)	Above normal (Ca >10.5 mg/dL)	P-value	χ²
Mild	1	7	2	0.001*	20.25
Moderate	20	12	0		
Severe	8	0	0		

A significant inverse relationship was observed between calcium levels and disease severity (p = 0.001), with mean calcium levels decreasing progressively from mild (9.01 ± 0.98 mg/dL) to moderate (7.71 ± 0.89 mg/dL) and severe cases (6.24 ± 0.65 mg/dL). Similarly, patients who died had significantly lower mean calcium levels (6.17 ± 0.67 mg/dL) compared to those who were discharged (8.08 ± 1.00 mg/dL; p = 0.001). Furthermore, patients requiring surgical intervention had lower mean calcium levels (6.89 ± 0.89 mg/dL) than those managed conservatively (8.30 ± 1.05 mg/dL; p = 0.005). These findings highlight the prognostic utility of serum calcium levels in assessing disease severity, predicting outcomes, and determining the need for surgical intervention in acute pancreatitis (Table 6).

**Table 6:** Association of calcium levels with CT Severity Index, outcomes, and intervention type. *Legend: Data analyzed using one-way ANOVA (severity index), independent t-test (outcomes and intervention type). p <0.05 indicates statistical significance.

Variable	Domain	Mean calcium	P-value	T statistics
Severity index	Mild	9.01 ± 0.98	0.001*	18.62
	Moderate	7.71 ± 0.89		
	Severe	6.24 ± 0.65		
Outcomes	Death	6.17 ± 0.67	0.001*	6.92
	Discharge	8.08 ± 1.00		
Intervention type	Conservative	8.30 ± 1.05	0.005*	3.00
	Surgical	6.89 ± 0.89		

A strong negative correlation was observed between serum calcium levels and both the duration of hospital stay (r = -0.694, p = 0.001) and BISAP scores (r = -0.684, p = 0.001), indicating that lower calcium levels were associated with longer hospitalizations and higher severity scores. Conversely, a strong positive correlation was found between BISAP scores and hospital stay duration (r = 0.820, p = 0.001), suggesting that patients with higher BISAP scores had prolonged hospital stays. These findings reinforce the role of serum calcium as a potential prognostic marker in acute pancreatitis (Table 7).

**Table 7:** Correlation of calcium levels with duration of hospital stay and BISAP score, and correlation between BISAP score and duration of hospital stay. *Legend: Data analyzed using Pearson’s correlation test. p <0.05 indicates statistical significance. BISAP: Bedside Index for Severity in Acute Pancreatitis

Variables	Correlation coefficient	P-value
Calcium and duration of hospital stay	-0.694	0.001*
Calcium and BISAP score	-0.684	0.001*
BISAP score and duration of hospital stay	0.820	0.001*

## Discussion

The pancreas has dual exocrine and endocrine functions. In acute pancreatitis, premature activation of pancreatic enzymes triggers inflammatory cascades that lead to autodigestion and varying degrees of tissue injury. While mild to moderate cases are often self-limiting, severe acute pancreatitis is associated with multi-organ failure and a substantially higher risk of morbidity and mortality. Consequently, early recognition and prompt management of severe disease are essential to improving clinical outcomes [10].

Hypocalcemia is frequently observed in critically ill patients and has been consistently linked to disease severity across diverse clinical conditions, including acute pancreatitis. Beyond its fundamental role in neuromuscular and cardiovascular function, serum calcium also serves as an indirect marker of nutritional status and systemic inflammation [11]. Mechanistically, hypocalcemia in acute pancreatitis is primarily attributed to fat saponification, wherein free fatty acids released by pancreatic lipase bind to calcium, forming insoluble soaps. Additional contributing factors include hypoparathyroidism, hypomagnesemia, and cytokine-mediated alterations in calcium metabolism [12].

This prospective observational study aimed to assess the utility of serum calcium levels as a prognostic marker in patients with severe acute pancreatitis. A total of 50 patients were enrolled. The mean age of participants was 37.92 ± 13.05 years, with a male predominance (66%), consistent with prior Indian and international studies [1,3-5,10,13]. Gallstones were the most common etiology (58%), followed by alcohol (30%), aligning with regional epidemiological patterns, where gallstones predominate in the West and alcohol in India [14].

Biochemically, lipase levels were elevated in 98% of patients (mean 1276.65 ± 526.27 U/L), further affirming its diagnostic significance. The mean serum calcium level was 7.73 ± 1.20 mg/dL, with 58% of patients exhibiting hypocalcemia (<8.5 mg/dL). Notably, all patients with severe disease (CTSI) had hypocalcemia, and lower calcium levels were significantly associated with moderate and severe disease categories (p = 0.001). These findings corroborate previous work by Wang et al., Bilgili et al., and others, who demonstrated a clear association between lower calcium levels and increased disease severity [1,4,5,10,13,15-18].

Calcium levels were inversely associated with BISAP scores (r = -0.684, p <0.001) and duration of hospital stay (r = -0.694, p <0.001), suggesting that lower calcium levels correlate with higher clinical severity and prolonged hospitalization. Additionally, BISAP scores were strongly correlated with longer hospital stays (r = 0.820), further validating its prognostic utility [5,14,19].

In our study, mortality was 18%, and all patients who died had significantly lower mean calcium levels (6.17 ± 0.67 mg/dL) compared to survivors (8.08 ± 1.00 mg/dL), supporting prior findings by Chhabra et al., Pokharel et al., and Peng et al., who identified hypocalcemia as an independent predictor of mortality in severe acute pancreatitis [1,3,4,20,21]. Furthermore, 40% of patients required surgical intervention, and these patients also had significantly lower calcium levels compared to those managed conservatively (6.89 ± 0.89 vs. 8.30 ± 1.05, p = 0.005), suggesting that hypocalcemia may also indicate a need for more aggressive management.

An important strength of this study is its prospective design, which allowed systematic data collection and reduced recall bias. Moreover, serum calcium is an inexpensive, easily available test, which makes our findings especially relevant for primary care and resource-limited settings where complex scoring systems may not always be practical. The study also correlated calcium levels with multiple prognostic indices, outcomes, and need for interventions, providing a comprehensive assessment of its clinical utility.

At the same time, there are certain limitations that must be acknowledged. The study was conducted at a single tertiary care center with a relatively small sample size, which may restrict the generalizability of the results. Another limitation is that we measured only total serum calcium; albumin-corrected calcium and ionized calcium, which are considered more accurate in critically ill patients, were not evaluated. Serial calcium measurements during hospitalization were also not performed, which could have provided additional insights into the dynamic relationship between calcium levels and disease progression.

Based on our observations, we recommend that larger multicenter studies be undertaken to validate the prognostic role of calcium in acute pancreatitis. Future research should include both albumin-corrected and ionized calcium, as well as serial monitoring, to better define their role in risk stratification. Integration of calcium measurements into existing prognostic scores may also be explored to enhance their predictive accuracy.

## Conclusions

This prospective study highlights the prognostic value of serum calcium levels in patients with acute pancreatitis. Hypocalcemia, observed in more than half of the patients, was significantly associated with increased disease severity, higher BISAP scores, prolonged hospital stays, greater need for surgical intervention, and higher mortality. A strong inverse correlation was found between serum calcium and both the CTSI and BISAP scores, reinforcing its utility as an early marker of poor prognosis. Given its simplicity, accessibility, and cost-effectiveness, serum calcium measurement within the first 24 hours of admission can serve as a valuable tool for early risk stratification and clinical decision-making in acute pancreatitis. Larger multicenter studies are warranted to further validate its role as an independent prognostic marker.
